# From Cholesterol Control to Critical Illness: A Case of Statin-Associated Rhabdomyolysis With Concurrent Acute Kidney Injury and Hepatotoxicity

**DOI:** 10.7759/cureus.86199

**Published:** 2025-06-17

**Authors:** Bola Habeb, Sandy Khair, Seth Fowler

**Affiliations:** 1 Internal Medicine, University of Florida College of Medicine, Ascension Sacred Heart, Pensacola, USA; 2 Radiation Oncology, Cairo University, National Cancer Institute, Cairo, EGY

**Keywords:** drug-induced toxicity, non-traumatic rhabdomyolysis, rhabdomyolysis causing acute kidney injury, side effects of statins, statin-induced hepatitis, statin-induced rhabdomyolysis

## Abstract

Statins, or HMG-CoA reductase inhibitors, are among the most commonly prescribed medications globally due to their well-established efficacy in lowering low-density lipoprotein cholesterol (LDL-C) and reducing the risk of atherosclerotic cardiovascular disease. Their widespread use in both primary and secondary prevention strategies has significantly contributed to declines in cardiovascular morbidity and mortality. Despite their favorable safety profile, statins are not without risk. Rare but potentially life-threatening complications such as rhabdomyolysis, hepatotoxicity, and acute kidney injury (AKI) can occur, particularly in individuals with specific risk factors, such as advanced age, renal impairment, high-dose therapy, or concurrent use of drugs that alter statin metabolism. Understanding the spectrum and mechanisms of these adverse effects is essential for prompt identification and management to prevent serious outcomes.

## Introduction

Statins (HMG-CoA reductase inhibitors) are widely prescribed lipid-lowering agents that play a pivotal role in the primary and secondary prevention of cardiovascular disease. While generally considered safe and well tolerated, statins can be associated with rare but serious adverse effects, including rhabdomyolysis, hepatotoxicity, and acute kidney injury (AKI) [1,2].

Rhabdomyolysis is the most severe form of statin-induced myotoxicity and is characterized by widespread skeletal muscle breakdown, markedly elevated serum creatine phosphokinase (CPK) levels, myoglobinuria, and risk of AKI [3]. The incidence is estimated at approximately 3.4 cases per 100,000 person-years, though the risk is higher with high-dose therapy, drug interactions (e.g., CYP3A4 inhibitors), renal impairment, and certain genetic predispositions [1,4].

In parallel, statin-induced liver injury is rare but can manifest as elevated transaminases, cholestatic or hepatocellular patterns of injury, and, in extreme cases, acute liver failure. Although asymptomatic aminotransferase elevation is more common and often transient, significant liver injury, especially in conjunction with rhabdomyolysis, is exceedingly rare [5].

When rhabdomyolysis leads to AKI, typically via myoglobin-induced tubular toxicity, the condition can progress rapidly and requires early recognition and aggressive management. The triad of statin-induced rhabdomyolysis, hepatotoxicity, and AKI is rare and poses a significant diagnostic and therapeutic challenge.

This case underscores an important knowledge gap in recognizing and managing rare but serious complications associated with long-term statin therapy. Although statin-induced rhabdomyolysis is a well-documented adverse effect, the concurrent occurrence of rhabdomyolysis, AKI, and hepatotoxicity remains exceedingly rare and underreported, particularly in patients without recent dosage changes. It challenges the prevailing assumption that statin toxicity predominantly arises early in treatment, highlighting the need for continued vigilance in long-term users, especially those with comorbidities such as chronic kidney disease. Furthermore, the case illustrates the absence of standardized monitoring protocols and predictive tools to identify individuals at higher risk, emphasizing the need for further research into effective risk stratification and early detection strategies in this population.

## Case presentation

A 71-year-old Caucasian male patient with a medical history of hypertension, hyperlipidemia, prediabetes, coronary artery disease with multiple coronary stents, and stage IIIb chronic kidney disease (with prior episodes requiring intermittent hemodialysis) presented with progressive generalized weakness. He had been in his usual state of health until five days prior to admission, when he began experiencing increasing weakness, ultimately requiring the use of a walker. On the day of presentation, he was unable to ambulate and nearly fell but was able to ease himself to the floor without injury. The patient reported a good appetite and adequate oral hydration. He denied recent trauma, vaccinations, or changes in his medication regimen. Of note, he had recently completed a course of cephalosporin prescribed by his primary care physician for a urinary tract infection. Laboratory studies performed 10 days earlier showed a creatinine level of 2.07 mg/dL, consistent with his baseline renal function.

The patient's home medications included aspirin 81 mg daily (delayed-release), calcitriol 0.25 mcg daily, clopidogrel 75 mg daily, empagliflozin (Jardiance) 25 mg each morning, finasteride 5 mg daily, metoprolol succinate 50 mg daily (extended-release), spironolactone 25 mg daily, and rosuvastatin 40 mg daily for the last 25 years.

Clinical findings

Physical examination revealed a temperature of 37.5°C, blood pressure of 132/78 mmHg, pulse of 93 beats per minute (bpm), 18 breaths per minute, and oxygen saturation of 98% on ambient air. The abdominal exam was normal, with a soft, lax abdomen and active bowel sounds. The cardiovascular exam was normal, with a regular rate and rhythm and no audible murmurs. Pulmonary examination demonstrated clear lungs to auscultation with no wheezing, rales, or rhonchi.

Diagnostic assessment

Laboratory results obtained on admission are demonstrated in Table 1. 

**Table 1:** The patient's laboratory data obtained on admission BUN: blood urea nitrogen; AST: aspartate aminotransferase; ALT: alanine aminotransferase; INR: international normalized ratio; TSH: thyroid stimulating hormone; pH: potential of hydrogen; pCO_2_: partial pressure of carbon dioxide; pO_2_: partial pressure of oxygen; A1C: glycated hemoglobin * Abnormal lab values.

Parameters	Patient's values on admission	Reference range, adults
Hemoglobin (g/dL)	14.6	12.0–15.5
Hematocrit (%)	44.3	34.9–44.5
White cell count (per mm3)	16900*	3500–10500
Platelet count (per mm3)	408,000	150,000–450,000
Sodium (mEq/dL)	136	135–145
Potassium (mEq/dL)	3.9	3.5–5.1
BUN (mg/dL)	67*	12–21
Creatinine (mg/dL)	3.59*	0.72–1.25
ALT (units/L)	338*	9–29
AST (units/L)	509*	12–31
Alkaline phosphatase (U/L)	350*	40-150
Total bilirubin (mg/dL)	1.1	0.2-1.2
INR	1	-
TSH (mcIU/mL)	2.23	0.35–4.9
pH	7.23*	7.31–7.41
PCO_2_ (mmHg)	45	35–45
PO_2_ (mmHg)	76	60-80
Bicarbonate (mEq/dL)	15*	22–29
Anion gap (mmol/L)	9	4-12
Blood glucose (mg/dL)	121	70-99
A1C	6.1	≤6.5

**Figure 1:**
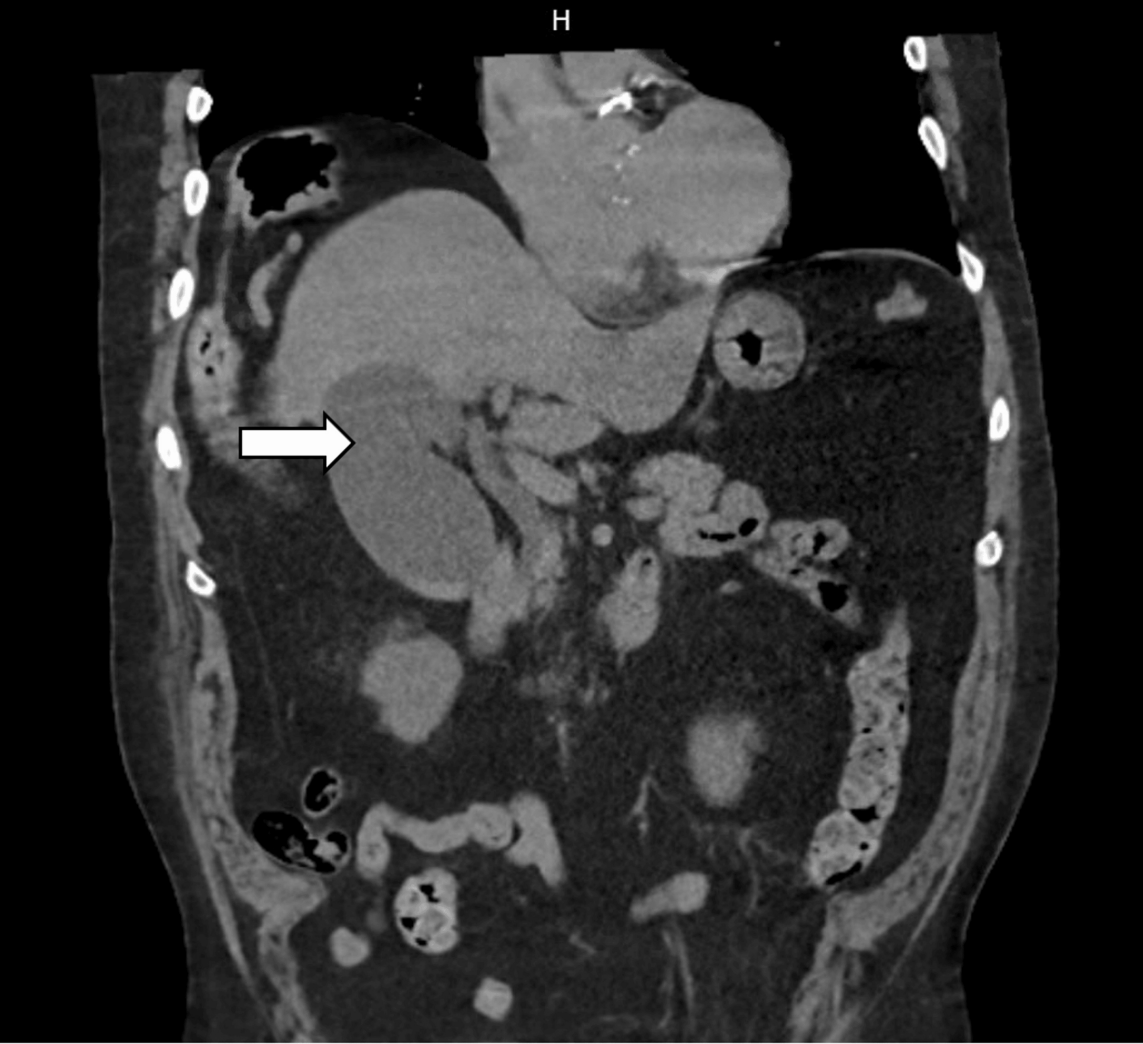
Non-contrast computed tomography (CT) of the abdomen and pelvis showing a distended gallbladder with gallstones.

Urinalysis demonstrated amber-colored urine with 100 mg/dL of protein, glucose >500 mg/dL, and microscopic hematuria with 22 red blood cells per high-power field (HPF). The urine drug screen was negative for methamphetamine, cocaine, opiates, phencyclidine, methadone, cannabinoids, barbiturates, and benzodiazepines. A non-contrast computed tomography (CT) scan of the abdomen and pelvis was unremarkable, except for a distended gallbladder containing multiple gallstones (Figure 1).

In light of the patient's clinical presentation with progressive generalized weakness and initial laboratory findings, including amber-colored urine, microscopic hematuria, AKI, and evidence of hepatotoxicity, further diagnostic evaluation was performed, as outlined in Table 2.

**Table 2:** Additional laboratory data CK: creatine kinase; Hep A IgM: hepatitis A virus Immunoglobulin M; Hep Bs Ag: hepatitis B virus surface antigen; Hep C Ab: hepatitis C virus antibody; Hep B core IgM: hepatitis B virus core antibody, Immunoglobulin M; HIV Ag, Ab: human immunodeficiency virus antigen, antibody; ANA: antinuclear antibody; ASMA: antismooth muscle antibody; AMA: antimitochondrial antibody * Abnormal lab values

Parameters	Patient's values	Reference range, adults
Total CK (IU/L)	15,643*	30-200
Hep A IgM	Nonreactive	-
Hep Bs Ag	Nonreactive	-
Hep C Ab	Nonreactive	-
Hep B core IgM	Nonreactive	-
HIV Ag, Ab combo screen	Nonreactive	-
Acetaminophen level (mcg/mL)	<3.0	<=30.0
Salicylate level (mg/dL)	<5.0	5-29
Ethanol (mg/dL)	<10	12–21
ANA	Negative	-
ASMA (units)	6	0-19
AMA (u/mL)	1.5	<=3.9

Given the patient’s presentation with progressive generalized weakness and markedly elevated CPK levels, in the context of prolonged high-intensity statin use and underlying chronic kidney disease, a diagnosis of statin-induced atraumatic rhabdomyolysis was established. Following a comprehensive evaluation that ruled out infectious, toxic, and autoimmune causes, the liver injury was also attributed to statin-induced hepatotoxicity as the most likely etiology.

**Figure 2:**
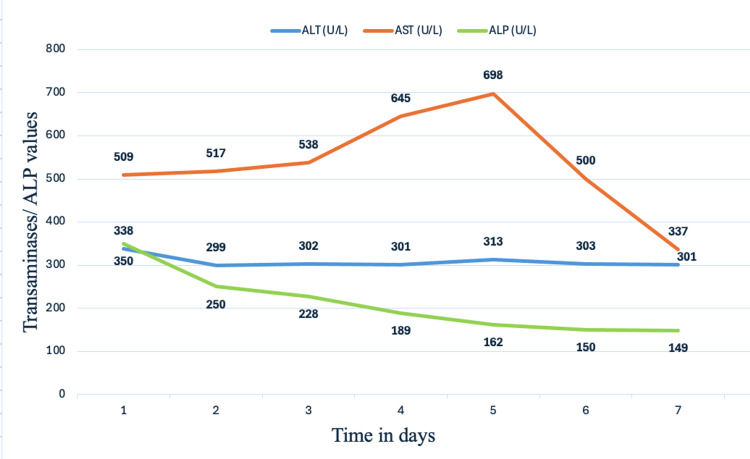
Graph showing transaminases and ALP trend over seven days ALT: alanine transaminase; AST: aspartate transaminase; ALP: alkaline phosphatase

**Figure 3:**
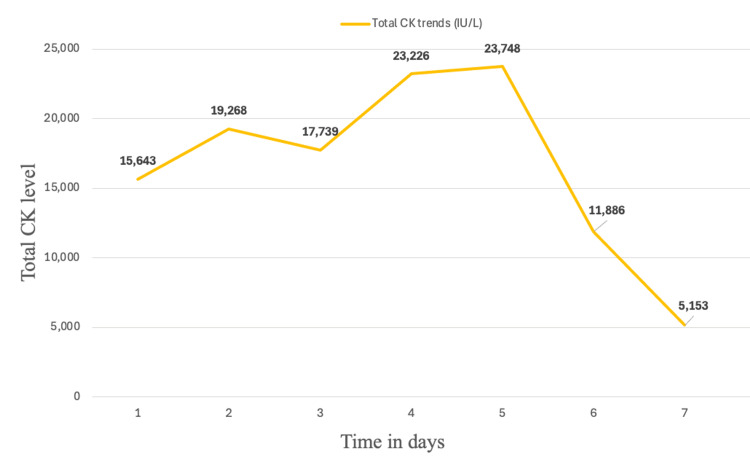
Graph showing total CK trends over seven days. CK: creatine kinase

Consequently, rosuvastatin was discontinued upon admission, and the patient was initiated on intravenous normal saline at a rate of 150 mL/hour. A follow-up arterial blood gas on hospital day two demonstrated resolution of the patient’s metabolic acidosis. Serial trends of aspartate aminotransferase (AST), alanine aminotransferase (ALT), alkaline phosphatase (ALP), and total creatine kinase (CK) levels throughout the patient’s seven-day hospitalization are illustrated in Figures 2, 3.

The patient’s overall condition showed marked improvement, and he was evaluated by the physical therapy team, who recommended discharge to a skilled nursing facility for continued rehabilitation. He was counseled on the importance of permanently discontinuing statin therapy, maintaining adequate hydration, and ensuring close follow-up with his primary care provider to monitor symptom resolution and normalization of laboratory values.

## Discussion

Lipid-lowering agents comprise a broad spectrum of pharmacologic therapies aimed at managing dyslipidemia and mitigating the risk of atherosclerotic cardiovascular disease. Statins remain the cornerstone of treatment, exerting their effects by competitively inhibiting HMG-CoA reductase, the rate-limiting enzyme in hepatic cholesterol biosynthesis, thereby increasing low-density lipoprotein (LDL) receptor expression and promoting the clearance of LDL cholesterol (LDL-C) from the bloodstream [6]. Additional therapeutic options include ezetimibe, which inhibits intestinal absorption of cholesterol; bile acid sequestrants, which interrupt enterohepatic circulation by binding bile acids in the gastrointestinal tract; PCSK9 inhibitors, which are monoclonal antibodies that enhance hepatic LDL receptor recycling and clearance; and fibrates, which primarily lower triglyceride levels through activation of peroxisome proliferator-activated receptor-alpha (PPAR-α) [6]. Niacin, although used less frequently due to its adverse effect profile, can reduce LDL-C and increase high-density lipoprotein cholesterol (HDL-C) [6]. The selection of lipid-lowering therapy is guided by individual lipid parameters, overall cardiovascular risk, tolerability, and the presence of comorbid conditions, with combination regimens often necessary for patients who do not achieve target lipid levels with monotherapy.

Common statins include atorvastatin and rosuvastatin, both high-potency agents capable of significant LDL-C reduction; atorvastatin is lipophilic and metabolized via CYP3A4, while rosuvastatin is more hydrophilic and primarily metabolized by CYP2C9, reducing drug interaction potential [7]. Simvastatin and lovastatin are moderate-potency, lipophilic statins also metabolized by CYP3A4, which may increase the risk of myopathy when used with certain other medications [7]. Pravastatin is a hydrophilic, moderate-potency statin with minimal cytochrome P450 metabolism, making it a safer option in patients with polypharmacy or liver concerns [7]. Fluvastatin, the least potent statin, is lipophilic and mainly metabolized by CYP2C9, offering a good safety profile for those needing modest LDL-C reduction [7]. Pitavastatin, a newer statin, is lipophilic with minimal CYP involvement and has shown favorable effects on HDL-C levels [7].

While generally well-tolerated, statins are not without adverse effects. Myotoxicity, ranging from mild myalgias to life-threatening rhabdomyolysis, is among the most serious complications, especially when associated with AKI and hepatotoxicity [1-3,7], as seen in this case.

Statin-induced rhabdomyolysis is a rare but well-documented adverse event, with an estimated incidence of 0.44 cases per 10,000 person-years in individuals receiving statin monotherapy [2]. While it most commonly occurs within the first six months of treatment initiation, delayed onset has also been reported, often triggered by factors such as acute illness, dehydration, or the introduction of interacting medications during long-term statin use. The underlying mechanism involves impaired hepatic uptake or metabolism of statins, leading to elevated plasma concentrations and direct skeletal muscle toxicity [3]. The risk is heightened with high-dose statins, concurrent use of CYP3A4 inhibitors, advanced age, renal impairment, and genetic factors such as SLCO1B1 gene polymorphisms [8]. Pathophysiologically, statins disrupt muscle cell membrane integrity and impair mitochondrial function, resulting in the release of intracellular contents, such as CK, myoglobin, and electrolytes, into the circulation. The presence of myoglobin in the urine (myoglobinuria) can lead to AKI through mechanisms including tubular obstruction, oxidative damage, and renal ischemia [2,3]. Clinical trial data report severe myopathy in approximately 0.08% of patients on lovastatin and simvastatin, while CK elevations exceeding 10 times the upper limit of normal have been observed in about 0.09% of those on pravastatin [4]. Although the overall risk of statin-associated myopathy is low across all approved agents, fatal rhabdomyolysis remains a rare complication, with fewer than one death per million prescriptions. Notably, cerivastatin, which has since been withdrawn from the market, demonstrated a substantially higher rate of fatal rhabdomyolysis, estimated to be 16 to 80 times greater than that of other statins [4].

Hepatotoxicity associated with statins is less common and usually manifests as transient elevations in transaminases that generally occur in 0.5% to 2.0% of cases and are dose-dependent [4]. True hepatocellular injury, particularly in the setting of rhabdomyolysis, may be secondary to the systemic inflammatory response and direct mitochondrial toxicity. The U.S. Food and Drug Administration (FDA) no longer recommends routine monitoring of liver enzymes in asymptomatic patients, though monitoring is indicated when symptoms suggest hepatic dysfunction [5].

In this case, the patient presented with generalized weakness, significantly elevated CK levels, AKI, and transaminitis in the absence of recent trauma. Given the patient's long-term use of high-intensity statin therapy for 25 years and underlying chronic kidney disease, the constellation of findings strongly supports statin-induced rhabdomyolysis complicated by renal and hepatic dysfunction. The prolonged exposure to statins, compounded by impaired renal function, may have increased the susceptibility to this adverse effect.

Prompt recognition and discontinuation of the offending agent are crucial. Management involves aggressive intravenous hydration to prevent further renal damage, correction of electrolyte abnormalities, and close monitoring of renal and hepatic function. In severe cases, renal replacement therapy may be required.

Clinicians must maintain a high index of suspicion for statin-induced rhabdomyolysis in patients presenting with muscle pain, weakness, dark urine, or systemic symptoms, particularly when accompanied by renal or liver dysfunction. Monitoring CK levels in symptomatic patients and assessing renal function and liver enzymes are essential to guide therapy. While routine CK screening is not recommended in asymptomatic individuals, baseline measurements may be helpful in high-risk patients [5].

Given the proven cardiovascular benefits of statins, the risk-benefit ratio typically favors continued use. However, this case underscores the importance of individualized therapy, patient education on early symptoms of toxicity, and vigilance for rare but serious adverse effects.

## Conclusions

This case underscores statins' potential to cause severe multisystem toxicity, including rhabdomyolysis, AKI, and hepatotoxicity, an exceptionally rare but life-threatening triad. It serves as a reminder that even widely used and generally well-tolerated medications like statins can result in serious complications, especially when patient-specific risk factors or drug interactions are present. Clinicians should maintain a high level of suspicion when patients on statin therapy develop systemic symptoms and promptly investigate for signs of myotoxicity and organ dysfunction.

Careful patient selection, dose adjustment based on comorbidities, and vigilance for drug interactions are essential preventive strategies. In addition, timely recognition and management, including statin discontinuation and supportive care, are vital to reversing the course of statin-induced organ injury. Continued awareness and reporting of such rare adverse events can contribute to improved clinical guidelines and safer prescribing practices.
